# Nursing Leadership Practices Among Nurse Managers in the Kumasi Metropolitan Area: An Exploratory Descriptive Qualitative Study

**DOI:** 10.1002/nop2.70791

**Published:** 2026-08-31

**Authors:** Emmanuel Obromah Amoh‐Kusi, Prosper Manu Abdulai, Collins Atta Poku, Victoria Bam

**Affiliations:** ^1^ School of Nursing and Midwifery, College of Health Science Kwame Nkrumah University of Science and Technology Kumasi Ghana; ^2^ 4 Medical Reception Station, Ghana Armed Forces Health Services 4BN, Uaddara Barracks Kumasi Ghana; ^3^ Department of Public Health Education, Faculty of Environment and Health Education University of Skills Training and Entrepreneurial Development Kumasi Ghana; ^4^ Department of Environmental Studies and Climate Change University of Liberia Fendell Campus Louisiana Montserrado County Liberia

**Keywords:** experiences, leadership practices, nurse manager, supervision, work environment

## Abstract

**Aim:**

To explore nurse managers' perceptions of how leadership practices contribute to the work environment within selected healthcare facilities in Ghana.

**Design:**

An exploratory descriptive qualitative design was employed to gain an in‐depth understanding of nurse managers' leadership experiences.

**Methods:**

Nineteen nurse managers with at least 2 years of managerial experience from three healthcare facilities in the Kumasi Metropolitan Area were purposively selected to participate in the study. Data were collected through semi‐structured in‐depth interviews and were analysed using thematic analysis.

**Results:**

Findings revealed that nurse managers commonly adopted a mixed (situational) leadership approach, adapting their leadership behaviours to meet varying workplace demands. Other leadership styles, including transformational, transactional, democratic, and laissez‐faire, were also reported. Effective communication, staff mentorship, and participatory decision‐making and supportive supervision emerged as key leadership practices that fostered a healthy work environment. Participants also identified organisational support, adequate staffing, and professional development opportunities as important enablers of effective leadership.

**Conclusion:**

Nurse managers described flexible, context‐responsive leadership practices involving supervision, communication, mentorship, participatory decision‐making, motivation, and adaptation to organisational demands. Participants perceived these practices as relevant to the nursing work environment, while staffing constraints, workload, resource limitations, and organisational structures were described as contextual challenges. Because the study reflects nurse managers' perceptions, the findings do not establish causal effects on staff retention, job satisfaction, workforce performance, or patient outcomes.

**Implications for Nursing Practice:**

Leadership education, structured mentorship, adequate staffing, and supportive organisational systems may be considered as potential areas for intervention and should be evaluated for feasibility and effectiveness in future implementation studies.

**No Patient or Public Contribution:**

No patient or public involvement in this study.

## 1 Introduction

The increasing complexity of healthcare delivery requires leadership approaches that effectively manage human resources, foster teamwork, and support high‐quality patient care. Within healthcare organisations, nurses constitute the largest professional group and maintain the most direct and sustained contact with patients (Poku et al. 2020; Aberese‐Ako et al. 2018). Consequently, nurses' work experiences and job satisfaction are closely linked to patient outcomes, organisational performance, and the overall quality of care delivery (Kokoroko and Sanda 2019; Kieft et al. 2014). Nurse managers encounter significant challenges in the performance of their day‐to‐day activities in a modern‐day rapidly changing work environment. The important role of integral leadership as a holistic leadership approach in nursing, which considers both intrinsic and extrinsic values of individual leaders, cannot be over emphasised (Choi and Cho 2025). Leadership practices within nursing have been widely recognised as a critical determinant of the work environment. Effective nursing leadership has been associated with improved job satisfaction, reduced burnout, enhanced teamwork, and better patient outcomes (Cummings et al. 2018; Aiken et al. 2013). In contrast, ineffective leadership contributes to high stress levels, low morale, and increased attrition among nurses (Buerhaus et al. 2017; Labrague et al. 2020; Labrague 2024). Contemporary nursing leadership literature emphasises leadership styles that foster empowerment, collaboration, and professional development, particularly in complex, resource‐constrained healthcare systems (Almutari and Almutairi 2023; Masike and Mahomed 2025).

Globally, transformational leadership has gained prominence in nursing for its focus on motivation, shared vision, and individualised consideration (Cummings et al. 2018; Bass and Avolio 1994). Studies from Europe and Africa have shown that transformational and participative leadership styles are associated with improved nurse satisfaction and safer patient care (Gardulf et al. 2016; Akintola and Chikoko 2016). However, nursing leaders continue to face challenges, including workforce shortages, limited resources, and increasing service demands, which constrain the consistent application of effective leadership practices (Cicolini et al. 2019; Mokoka et al. 2020).

In Ghana, the nursing workforce operates within a context characterised by high workloads, staff shortages, resource limitations, and complex patient needs (Poku et al. 2020; Aberese‐Ako et al. 2018). A lot of barriers including individual inefficiencies, institutional bottlenecks, and policy inconsistencies have been identified as major barriers in the effective implementation of nurse managers' leadership practices (Zhao et al. 2026). However, effective leadership improves the quality of care for patients, their safety, and enhances team performance; clinical experience alone is not sufficient to guarantee good results, especially in a low‐resource setting without the much‐needed leadership experience (Martinez and Zuniga 2026).

Although several policy documents have been developed to improve working conditions for healthcare workers (Ghana, 2013; MOH, 2020; National Health Policy, 2020), evidence regarding the implementation and effectiveness of leadership practices within clinical nursing environments remains limited. Existing studies have primarily focused on nursing education and management structures, with less attention given to leadership practices as experienced by nurses in everyday clinical practice (Heinen et al. 2019; Keyko et al. 2016; Scully 2015).

Although previous studies have examined nursing leadership styles and their associations with job satisfaction, organisational performance, and patient outcomes, most have relied on quantitative designs and have focused primarily on identifying dominant leadership styles rather than understanding how nurse managers enact leadership in everyday clinical practice. Moreover, qualitative evidence describing how leadership practices contribute to creating healthy work environments within the Ghanaian healthcare context remains limited. The Frederick Herzberg two‐factor theory of motivation and hygiene factors, which has a direct correlation on job satisfaction and dissatisfaction while ultimately influencing the work environment of an institution, was adopted to address the gaps in the study. Addressing this gap is important for informing leadership development and organisational strategies that support nursing practice. Therefore, this study explored the leadership practices employed by nurse managers and how these practices influence the creation of a healthy work environment in selected healthcare facilities in the Kumasi Metropolitan Area.

## 2 Methods

### 2.1 Study Design

An exploratory, descriptive, qualitative research design was employed to examine nursing leadership practices among nurse managers (Hunter et al. 2019). Exploratory descriptive qualitative approaches are widely used in health and nursing research because of their flexibility and ability to capture participants' experiences in their own words (Hunter et al. 2019). This design was appropriate for exploring leadership practices, their perceived impacts, and the contextual factors influencing their implementation in clinical nursing settings. The study was conducted and reported in accordance with the Consolidated Criteria for Reporting Qualitative Research (COREQ) guidelines provided as Table S1.

### 2.2 Study Setting

The study was conducted in the Kumasi Metropolitan Area, located in the Ashanti Region of Ghana. Healthcare services in the metropolis are provided by government, quasi‐government, and private healthcare institutions. These facilities provide a wide range of healthcare services, including public health services, maternal and child health care, emergency care, medical and surgical services, and specialised clinical care. Three public healthcare facilities located within the Kumasi Metropolitan Area were purposively selected for the study. These facilities were chosen because they have established organisational structures for nursing leadership, relatively stable nurse‐to‐patient ratios, and sufficient numbers of nurse managers capable of providing rich information relevant to the study objectives.

### 2.3 Study Population

The study population comprised nurse managers working at the three selected healthcare facilities in the Kumasi Metropolitan Area. Nurse managers were selected because they hold leadership and supervisory responsibilities within clinical units and are directly involved in decision‐making, staff supervision, and promoting healthy work environments in healthcare settings.

#### 2.3.1 Eligibility Criteria

Eligible participants were nurse managers who had served in a managerial role for at least 2 years, had worked at the facility for at least 6 months, were present during the data collection period, and provided written informed consent to participate in the study. Nurses who were on rotation, had worked in the facility for less than 6 months, were absent during the data collection period, or declined to participate were excluded.

### 2.4 Sampling Technique and Sample Size

Purposive sampling was used to recruit nurse managers with relevant experience in nursing leadership practices. Potential participants were identified through the nursing administration of the selected healthcare facilities. A total of 25 nurse managers were approached for participation; of these, 19 participated in the interviews, while 6 declined participating for no specific reason given.

The sample size was determined by data saturation, defined as the point at which no new themes, categories, or meaningful insights emerged from successive interviews. During data collection and concurrent analysis, the research team reviewed interview transcripts after each interview and compared emerging codes and themes with those identified previously. Initial thematic saturation was observed after the 17th interview, as no additional codes or concepts emerged. Two further interviews (18th and 19th) were conducted to confirm that thematic saturation had been reached and that no new information was generated. Consequently, data collection was discontinued after the 19th interview, as the findings were considered sufficiently rich and comprehensive to address the study objectives. The demographic profile of participants is reported in Table 1.

**TABLE 1:** Characteristics of the nurse manager participants (*N* = 19).

Demographics	Description	Frequency (nurse managers)
Total number of respondents	Nurse managers	19
Facilities	4 Medical Reception Station	5
	Kumasi South Government hospital	7
	Suntreso Government Hospital	7
Years of service	2–5 years	2
	6–10 years	12
	Above 10 years	5
Years in management	2–5 years	7
	6–10 years	10
	11– above	2

### 2.5 Data Collection

Following ethical approval from the Committee for Human Research, Publication and Ethics (CHRPE) of Kwame Nkrumah University of Science and Technology (KNUST) (Approval No. CHRPE/AP/148/24), granted on 4 March 2024, and approval from hospital management, eligible nurse managers were approached in person by the researchers. Data collection was conducted from 5 April 2024 to 30 June 2024. The researchers explained the study objectives, procedures, and voluntary nature of participation to eligible participants. Interested individuals received a participant information sheet and were invited to participate. Written informed consent was obtained before the interviews. Interviews were scheduled at times convenient for participants to minimise disruption to their professional responsibilities.

Data were collected using a semi‐structured in‐depth interview guide. The semi‐structured interview guide was developed following a review of the literature on nursing leadership, healthy work environment, and Herzberg's Two Factor Theory, together with the study objectives. The guide consisted of open‐ended questions designed to explore participants' leadership practices, leadership styles, perceived facilitators and barriers, and their experiences in promoting a healthy work environment. The interview guide was reviewed by qualitative research experts and pilot‐tested with two nurse managers from a healthcare facility not included in the study to assess the clarity, relevance, and sequencing of the questions. Minor revisions were made to improve the questions' wording and flow prior to data collection.

The interview guide explored key topics, including as follows:

- leadership practices among nurse managers
- decision‐making processes
- supervision and mentoring of nursing staff
- staff motivation strategies
- challenges encountered in creating a healthy work environment

Examples of interview questions included:

- How would you describe your experience in nursing practice and leadership?
- What does nursing leadership mean to you in your current role?
- How do you supervise and support nurses in your unit?
- What strategies do you use to motivate nursing staff?
- Can you describe situations where your leadership contributed to a positive or negative work environment?
- What challenges do you encounter when trying to create a healthy work environment for nurses?

Probing questions were used to encourage participants to elaborate on their responses and provide specific examples. Interviews were conducted face‐to‐face in private offices or quiet spaces within the health facilities to ensure confidentiality and minimise interruptions. Each interview lasted approximately 30–45 min. With participants' consent, all interviews were audio‐recorded and supplemented with field notes documenting contextual observations and non‐verbal cues. No repeat interviews were conducted. Interview recordings were securely stored and transcribed verbatim. Transcripts were de‐identified using participant codes before analysis and imported into NVivo for data organisation and coding. Audio files, transcripts, field notes, and analytical materials were stored in password‐protected files accessible only to authorised members of the research team.

The semi‐structured interview guide was informed by Herzberg's Two‐Factor Theory. Questions explored participants' experiences of leadership practices and workplace conditions, including motivation, recognition, professional development, responsibility, supervision, interpersonal relationships, organisational policies, working conditions, workload, and resource constraints. The theory served as a sensitising framework for developing the interview questions while allowing participants to describe experiences and issues beyond the predetermined theoretical concepts.

### 2.6 Data Management and Analysis

Data were analysed using reflexive thematic analysis following Braun and Clarke's approach. The analysis was situated within a contextualist epistemological position, recognising that participants' accounts reflected their perceptions and experiences of leadership within particular organisational and healthcare contexts. The analysis therefore sought to interpret how nurse managers understood and described their leadership practices, leadership approaches, and workplace experiences rather than to establish objective causal effects.

The analysis followed the six phases of reflexive thematic analysis: familiarisation with the data, generation of initial codes, searching for potential themes, reviewing themes, defining and naming themes, and producing the final report. Transcripts were read repeatedly to develop familiarity with the dataset before initial codes were generated. Related codes were subsequently examined for patterns and grouped into potential themes. Themes were reviewed against the coded extracts and the full dataset and were refined to ensure coherence and distinction between themes.

NVivo was used to organise the transcripts, manage codes, retrieve coded data, and support the iterative development and review of themes. Coding and theme development were iterative rather than based on numerical measures of inter‐coder agreement.

Herzberg's Two‐Factor Theory informed interpretation rather than functioning as a rigid deductive coding framework. Initial coding remained grounded in participants' accounts, while emerging codes and themes were subsequently considered in relation to the theory's motivational and hygiene dimensions. Accounts concerning recognition, professional development, responsibility, advancement, and motivation were considered in relation to motivational factors, whereas accounts concerning working conditions, organisational policies, supervision, interpersonal relationships, staffing, workload, and resource constraints were considered in relation to hygiene factors. Leadership practices and leadership styles were retained as distinct analytic concepts where they did not directly correspond to either category.

### 2.7 Trustworthiness

The trustworthiness of the study was established using the criteria of credibility, dependability, confirmability, and transferability. Credibility was enhanced through transcript checking, whereby all 19 participants reviewed their interview transcripts for accuracy. This process was limited to verification of the accuracy of the transcribed interview content and did not involve participant review or validation of the study findings or themes. Participants did not request substantive changes to the transcripts. Dependability was strengthened by maintaining an audit trail documenting methodological decisions, coding procedures, and theme development throughout the study. Confirmability was promoted through researcher reflexivity, whereby the researchers continuously reflected on their assumptions and potential biases during data collection and analysis to minimise their influence on interpretation. Transferability was supported by providing detailed description of the study setting, participant characteristics, sampling procedures, and analytical processes to enable readers to determine the applicability of the findings to similar contexts.

### 2.8 Research Team and Reflexivity

The interviews were conducted by two research assistants, who have a Master of Philosophy in Nursing. Both interviewers had training and experience in qualitative health research and qualitative interviewing. Before data collection, the researchers received refreshers training in qualitative interviewing techniques and ethical conduct of research. The interviewers had no prior supervisory, managerial, clinical, or personal relationship with the participants.

Throughout the study, the researchers maintained reflexive notes to document personal assumptions, methodological decisions, and observations during data collection and analysis, thereby enhancing transparency and reducing potential researcher bias.

## 3 Results

### 3.1 Participant Characteristics

A total of 19 nurse managers, including the various unit managers from the three study sites, participated in the study. Participants were drawn proportionally from the three selected healthcare facilities. Nurse managers represented a range of professional grades, including Nursing/Midwifery Officers, Senior Nursing/Midwifery Officers, and Principal Nursing/Midwifery Officers and above. Most nurse managers had more than 6 years of clinical experience, with the majority having 6 to 10 years of management experience. Educational backgrounds included diplomas and degrees, with degree holders forming the majority.

### 3.2 Presentation of Themes and Sub‐Themes

Thematic analysis identified seven interrelated themes describing the leadership practices employed by nurse managers and the organisational factors influencing the creation of a healthy work environment (Table 2). To improve conceptual clarity, the themes are presented according to their distinct functions within nursing leadership. Supervision, communication, and professional development describe the day‐to‐day leadership practices undertaken by the nurse managers; leadership styles represent the broader approaches through which these practices are implemented. Motivation, organisational structure and workload represent contextual factors that either facilitate or constrain effective leadership and the development of a healthy work environment.

**TABLE 2:** Summary of themes, sub‐themes and representative quotations.

Themes	Sub‐theme(s)	Representative quotation
Supervision	Monitoring Staff	As a nurse manager, I supervise staff to ensure protocols are followed and quality care is maintained
	Delegation	
	Quality Assurance	
Communication	Open Communication	Communication is the foundation of leadership because it promotes trust and teamwork
	Feedback	
	Team Engagement	
Professional development	Mentorship	I mentor junior nurses to help them develop professionally and build confidence
	Coaching	
	Capacity Building	
Leadership styles	Mixed (Situational)	I don't rely on one leadership style: I adapt depending on the situation
	Democratic	
	Autocratic	
	Laissez‐Faire	
Motivation	Recognition	Motivating staff improves morale and encourages them to perform better
	Staff Encouragement	
Organisational structure	Reporting Lines	A clear reporting structure helps us manage staff effectively
	Institutional Support	
Workload	Staff Shortages	High workload limits our ability to supervise staff as effectively ae we would like
	Time Constraints	

### 3.3 Concept of Nurse Leadership Practices

#### 3.3.1 Presentation of Themes and Sub‐Themes

Table 3 presents the themes and sub‐themes under each theme used in the discussion, as a reference.

**TABLE 3:** Themes and sub‐themes for nurse managers.

Themes	Sub‐Themes
1. Supervision	Monitoring and evaluating staff activities
	Assignment of tasks and ensuring quality of service
2. Communication	Open communication
	Good interpersonal skills
3. Professional development	Training sessions and seminars
	Mentorship
4. Leadership styles	Mixed method
	Democratic
	Autocratic
	Laissez‐Faire
5. Motivation	Financial reward/citation
	Promotion and upgrading
	Addressing grievances
6. Organisational structure	Hierarchical structure
7. High workload	Staff strength
	Technology

### 3.4 Theme 1: Supervision

Supervision emerged as a core leadership practice through which nurse managers monitored staff performance, delegated responsibilities, ensured adherence to clinical standards, and supported professional development. Unlike leadership style, which reflects the overall approach adopted by managers, supervision refers to the day‐to‐day managerial activities used to maintain quality care and staff accountability. Most interviewees believed that nursing leadership involves an experienced person directing, teaching, and ensuring that an inexperienced person does the right thing to achieve optimal goals and ensuring staff follow due procedures and protocols. These responsibilities included delegating specific tasks to individuals to build their capacity to take on future roles. It also involved setting up teams for specific activities or projects to encourage the desire and willingness to work collaboratively to achieve the organisational goal. When used by a nurse manager, all these qualities will ensure the quality and consistency of service is delivered at the facility.

#### 3.4.1 Sub‐Theme 1: Monitoring and Evaluating Staff Activities

Regarding supervision in nursing leadership, participants believed that interventions such as monitoring by senior members of the profession and evaluating activities assigned to juniors help enhance their competencies. The quotes below from some nurse managers support this sub‐theme.

Nurse Leaders are to monitor the quality of service by subordinates. (S1P‐1)

Taking charge of subordinates and ensuring that work is done efficiently and effectively, observing and directing tasks assigned to subordinates for good execution and training. Critiquing and taking responsibility for subordinates are responsibilities of nurse managers. (S2P‐2)

I try to allocate time specifically for team discussions and feedback sessions, even if it means being more efficient in other areas. It's about finding a balance and prioritising the involvement of my team in the decision‐making process. (S3P‐3)

#### 3.4.2 Sub‐Theme 2: Assignment of Task and Ensuring Quality of Service

Assigning specific tasks to individuals and teams is one tool recommended by nurse managers to achieve daily objectives and ensure everyone is involved in the unit's affairs. It helps build the confidence of the less experienced and encourages knowledge sharing. However, the nurse managers suggested that these assignments should not be instructed in a way that makes it seem like a punishment.

When instructing nurses under my supervision, I ensure that my instructions are full and clear before I leave. If you don't understand anything, I make sure you do. If you don't do this, mistakes will be made under your watch (S3P‐5)

When you give instructions without ensuring everyone has understood, especially with general nurses, it can be fatal. Nurses work under a lot of pressure, and so it is easy for instructions to be forgotten or misunderstood if you are not clear in giving them (S2P‐1)

Conversely, some participants disagreed with this form of supervision. Some of the participants suggested an autocratic form of supervision.

I am an autocratic leader. When you are a leader, you make sure that when you are working, you put yourself in. And if anything is wrong, when you advise them, they will listen (S3P‐5)

### 3.5 Theme 2: Communication

Communication emerged as a distinct leadership practice centred on information exchange, active listening, interpersonal relationships, and staff engagement. While supervision focused on directing and monitoring staff activities, communication facilitated collaboration, trust, and participatory decision‐making within clinical teams. Effective communication was identified as a fundamental component of nursing leadership. Regular meetings, suggestion boxes, and open‐door policies, along with in‐service training, create an environment where staff feel comfortable sharing their ideas and opinions and learning from colleagues and seniors.

#### 3.5.1 Sub‐Theme 1: Open Communication

A nurse manager at the study Site 1 (S1) during the one‐on‐one interview said

Communication is key. It's not just about talking, but truly listening and understanding what your team needs. (S1P‐3)

In the interview, a nurse manager from study Site 3 (S3) also added:

A leader must be an excellent communicator. This means being approachable, open, and clear in your expectations. (S3P‐3)

It's all about team building and at the end of the day you should be able to carry everyone along as a leader by listening to all their concerns. (S3P‐3)

Also, a respondent from study Site 2 (S2) responded that:

Effective communication builds trust and ensures that everyone is on the same page, which is vital in a healthcare setting. (S2P‐2)

#### 3.5.2 Sub Theme 2: Good Interpersonal Skills

In communication, participants agreed that when presenting instructions, one should be circumspect and take note of both verbal and non‐verbal cues. Presenting instructions can be a bit complicated because individuals perceive things differently. It is therefore important to consider how instructions are communicated to reduce the risk of misinterpretation and confusion and achieve effective communication. The following quotes indicate participants' views on the importance of interpersonal skills in leadership practice.

I actively work on building trust within my team. By fostering an environment where nurses feel safe to express their opinions without fear of negative consequences, I can encourage more open and honest communication. (S2P‐4)

Having a diverse and inclusive team can also facilitate democratic leadership. When team members come from different backgrounds and have varied experiences, it enriches the decision‐making process and promotes a culture of inclusivity and collaboration. (S1P‐3)

Effective communication channels are crucial. When there is a culture of open and transparent communication, especially if you, the manager, have a good interpersonal relationship with your staff, it becomes much easier for subordinates to reach out to you, the manager (S1P‐2)

### 3.6 Theme 3: Professional Development

Interviewees underscored the role of nurse leaders in fostering professional growth and providing mentorship opportunities to subordinates. These included organising in‐service training sessions, seminars, mentorship, and upgrading personnel to meet emerging professional standards.

#### 3.6.1 Sub‐Theme 1: Training Session, Seminars and Mentorship

Interviewees reiterate that nurses need frequent training and seminars to keep them up to date on the profession's progress. Consistent training and seminars will benefit nurses in delivering quality healthcare.

Nurse leaders must invest in their team's development, helping them to build their skills and advance their careers. (S2P‐2)

Mentorship is a cornerstone of nurse leadership. It's about guiding your colleagues, providing feedback, and supporting their journey. (S1P‐3)

Professional growth should be encouraged. Leaders need to create opportunities for their team to learn and develop new skills. (S3P‐3)

I believe that we have so much to learn in our profession. Human healthcare is always changing, and as such, we need to constantly upgrade our knowledge if we want to serve our patients diligently (S2P‐3)

### 3.7 Theme 4: Leadership Styles

Unlike the preceding themes, which described specific leadership practices, this theme focuses on the overarching leadership approaches adopted by nurse managers when interacting with staff. Participants emphasised that no single leadership style was appropriate for all situations; instead, they adapted their leadership approach according to contextual demands and organisational needs. Other alternatives cited by nurse managers as their preferred choice included transformational, transactional, crisis, presence, self‐efficacy, situational, and participative styles of leadership. However, more emphasis was placed on the four significant sub‐themes that were identified.

#### 3.7.1 Sub‐Theme 1: Mixed (Situational) Leadership Approach

Many nurse managers reported adopting a mixed or situational leadership approach, adapting their leadership behaviours to the demands of specific situations.

As a nurse manager, if you are too autocratic or democratic, your nurses do not always respond to you appropriately as you will wish, so I prefer the mixed method style. (S2P‐4)

I wouldn't say I use democratic, autocratic or any other because it depends on the situation, and sometimes, I even use crisis leadership as an emergency nurse. (S3P‐3)

#### 3.7.2 Sub‐Theme 2: Democratic Leadership

Some participants noted that they prefer to use the democratic leadership style, prioritising collaboration and shared decision‐making. They value input from team members and foster a participative environment. Participants felt respected and more involved in the decision‐making process.

And when you are open, people come to you willingly to share ideas, so you have to be free and firm in your decisions so that people can easily approach you. (S2P‐5)

In fact, as I said, I see nursing to be a team effort. You know, at the end of the day, it's the patient's health care that we are all looking at, but that should not put… Regardless of that, you shouldn't let it influence you, the health personnel, let's say, the health team. So, I always want to employ a democratic way of leadership whereby you bring up something, then you deliberate it with either your superiors or your junior ones, okay, so that at least you guys can reach an amicable solution to it (S1P‐1)

#### 3.7.3 Sub‐Theme 3: Autocratic Leadership

Some participants disagreed with the democratic form of supervision, citing delays and time constraints as reasons they sometimes prefer the autocratic style of leadership; as a result, some participants suggested an autocratic form of supervision.

I am an autocratic leader. When you are a leader, you make sure that when you are working, you put yourself in. And if anything is wrong, when you advise them, they will listen (S3P‐5)

When you try to be autocratic, you know, staff, nursing staff, what they can do there will be rumour mongers among us, people will be bringing you down when you want to bring things up. (S2P‐2)

#### 3.7.4 Sub‐Theme 4: Laissez‐Faire Leadership

Some nurse managers indicated a preference for a laissez‐faire approach, offering minimal direct supervision and allowing nursing staff to make decisions.

As a nurse manager, you sometimes trust your subordinates without always micromanaging everything that they do; it will not help them build their self‐confidence. (S3P‐4)

### 3.8 Theme 5: Motivation

Nurse managers highlighted the need for motivation in various forms to help nurses put out their best. Participants indicated that staff motivation enhances team morale and contributes to a positive work environment. They indicated that motivation can take various forms, including financial rewards, recognition for excellence, promotion and advancement, and other incentives such as awards and opportunities for professional development for those who excel in their various capacities.

#### 3.8.1 Sub‐Theme 1: Financial Reward/Citation

Participants cited financial motivation and citation as an essential element in motivating them to create a healthy working environment, a nurse manager at study Site 2 said.

Monetary reward is good, but not always in terms of monetary aspects. Like for example, today I am in the emergency unit, they can organise some brunch for the staff, some lunch, that's fine, that is also a form of motivation, oh yes and also best nurse at the facility (S2P‐3)

Nurse managers from other study sites reiterated that both intrinsic and extrinsic motivation catalyse their work.

As I said, motivation on the part of the nurses is one factor, and you cannot relegate financial motivation to the back bench even though they sometimes give some at the end of the year, but I think it's woefully inadequate, especially when you want to bring out the best in the manager and the nurses. (S3P‐1)

We simply don't have enough resources to provide meaningful rewards consistently, which undermines the effectiveness of the transactional approach. Financial constraints are a significant barrier. Without an adequate budget, it's hard to implement any incentive programs. Many of our staff are more motivated by intrinsic factors, such as personal growth and job satisfaction, rather than external rewards. In high‐stress environments, staff burnout is common, and the reward‐punishment model can sometimes exacerbate this issue. (S3P‐3)

#### 3.8.2 Sub‐Theme 2: Promotion and Upgrading

Promotion and upgrading were key motivators for nurse managers, and most considered them a form of motivation rather than professional development

I've been able to grow professionally because my leaders actively supported my career advancement, and I felt empowered to make clinical decisions, knowing my leaders trusted my judgment and skills. (S3P‐4)

I feel more comfortable and confident in my work when there's a clear structure and order to follow. The routines and protocols make our work environment more predictable and less chaotic, especially when it comes to promotion. I know that when I do my work diligently, and it's time for me to be promoted, sometimes you see the promotion interview as a mere formality (S1P‐4)

#### 3.8.3 Sub‐Theme 3: Addressing Grievances

Addressing grievances and creating the enabling environment for nurses to voice out their concerns was also a significant factor which nurse managers cited as a condition that creates a healthy work environment for them; they indicated that when you have a superior who is ready to listen not criticise all the time, you're always more than willing to voice out your concerns and even seek their opinion on other matters.

When everyone contributes to care plans and decisions, we provide better care because we're all on the same page and motivated to do our best. The different perspectives help find better solutions to problems. It's a more effective way to address patient needs. However, reaching consensus can take a long time, which can be frustrating when we're dealing with urgent situations. Yes, it's great that everyone gets a say. However, it can also lead to disagreements and conflicts that need to be managed carefully. (S2P‐3)

### 3.9 Theme 6: Organisational Structure

The study's findings indicate that organisational structure and long‐term culture are instrumental in helping nurse managers achieve their personal and organisational goals. Interviewees cited the organisational hierarchical structure, open channels of communication, feedback mechanisms, and a supportive and inclusive culture as making it easier for them to fit into the organisation and to give their utmost best in leading their subordinates in their various units as managers to achieve a healthy work environment.

#### 3.9.1 Sub‐Theme 1: Hierarchical Structure and Culture

A well‐defined organisational hierarchy facilitated effective reporting and communication.

The absence of these structures sometimes makes it difficult to properly report incidents and other work‐related matters, which can cause friction among staff and managers.

Support from upper management is essential. When the higher‐ups support and encourage a good nursing leadership approach, it legitimises and strengthens our efforts to engage staff in decision‐making processes. (S2P‐7)

I actively work on building trust within my team. By fostering an environment where nurses feel safe to express their opinions without fear of negative consequences, I can encourage more open and honest communication. (S3P‐6)

### 3.10 Theme 7: High Workload

Regarding high workload, nurse managers indicated that staff shortages have been a major problem for them over the years. Even though staff strength has increased from time to time, the significant increase in patient attendance over time has rendered it insignificant. However, the introduction of the electronic patient records system has reduced paperwork burden and improved the work environment.

#### 3.10.1 Sub‐Theme 1: Staff Strength

Staff shortages were identified as a major challenge affecting nurse managers' ability to maintain a healthy work environment. They cited burnout among both managers and nurses as a consequence of staff shortages, which sometimes makes it difficult to plan appropriately with the staff they have, knowing that some circumstances are likely to leave some of the staff indisposed at some point

Time constraints are a major issue, especially with the shortage of staff all the time and the frequent rotation of staff from one ward to another. (S2P‐2)

…even though this is an emergency unit you will realise that if I show you the duty roster you will find only few emergency nurses on the list and that means we have to constantly be training people on the job and by the time they are almost through with the training then they will be sent to another ward, however our emergency physician has made a case for us so I think for now they allow 2 people to go for the emergency course every year. (S3P‐2)

#### 3.10.2 Sub‐Theme 2: Technology

Participants noted that technology is a powerful enabler for nurse leadership, helping to overcome traditional barriers by enhancing communication, education, administrative efficiency, decision‐making, patient care, collaboration, and research. Nurse leaders who effectively leverage these technologies reported significantly improved healthcare outcomes and more efficient team leadership.

I leverage technology to facilitate democratic leadership. Online surveys, group chats, and collaborative platforms can make it easier to gather input and keep everyone involved, even when face‐to‐face meetings are not possible. (S3P‐2)

One of the biggest barriers to democratic leadership is time. Technology helps streamline many administrative tasks, freeing up time to engage with nursing staff. For instance, automated scheduling systems reduce the time spent on creating rosters manually, allowing for more time to be spent on team‐building and problem‐solving activities. (S3P‐1)

We use video conferencing tools for virtual meetings, which is especially useful for including night shift nurses who might otherwise feel left out of the decision‐making process. This way, everyone can participate, regardless of their shift. (S2P‐3)

## 4 Discussion

The study explored the leadership practices employed by nurse managers and examined how these practices contribute to creating a healthy environment. The results of the study indicated that participants' understanding of the concept of nurse leadership was that a leader is a supervisor who communicates effectively and has interpersonal skills. The nursing managers cited many leadership styles as their preferred choices, including transformational, transactional, crisis, presence, self‐efficacy, situational, and participative styles. However, the four sub‐themes that were significant across the study were mixed (situational), democratic, autocratic, and laissez‐faire leadership approaches. Cömert et al. (2016) reported that communication competence is a prerequisite for the quality of health care provided. The role of communication in promoting organisational efficiency is increasingly recognised and emphasised (Almatrooshi et al. 2016). The absence of effective communication can compromise patient safety and quality of care, so it is the responsibility of nurse managers to ensure effective communication and develop and maintain communication skills and channels in the clinical setting (Kwame and Petrucka 2020). Raeissi et al. (2023) reported that nurse leaders' communication skills, their ability to listen, articulate expectations clearly, and provide feedback have a significant impact on nurses' job satisfaction. Effective supportive communication in the nursing environment is an essential tool; according to Riley (2015), it promotes nurses' dignity and respect and is critical for building trust.

Several studies confirmed a connection between the leadership and communication skills of nurse leaders and their effect on achieving a healthy work environment (Specchia et al. 2021; Morsiani et al. 2017; Jankelová and Joniaková 2021). In turn, this is reflected in the patient's perception of the level of quality of healthcare (Specchia et al. 2021; Morsiani et al. 2017). The main predictors of a healthy work environment for nurses include a strong supervisory leadership style, degree of responsibility, decision‐making autonomy, and open communication by first‐line nurse managers (Albagawi 2019; Penconek et al. 2021). The study's outcome also aligns with that of (Peltonen et al. 2019), who reported that nurse managers are responsible for day‐to‐day operations in nursing departments and for supervising department staff. Peltonen et al. (2019) noted, however, that leaders typically supervise nursing teams and ensure the unit or hospital's overall success. Labrague also reported that nurse leaders oversee nursing units, ensuring that nurses follow established protocols and procedures that maintain patient safety and high‐quality care (Labrague 2024). Similarly, Wei et al. (2019) noted that nurse leaders must effectively communicate information about care practices, departmental needs, and organisational policies and issues. In clinical settings, they communicate treatment plans, diagnoses, and test results to patients and other healthcare providers. They also collaborate with doctors, pharmacists, social workers, and other nurses to implement evidence‐based care.

Nurse leaders build trusting, collaborative relationships with nursing staff, patients, and other healthcare leaders. This requires demonstrating empathy, asserting opinions in non‐judgmental ways, accepting and offering criticism constructively, and meeting promises and commitments (Kowalski et al. 2020).

In the current study, participants also indicated that nursing leadership is about managers advocating for their staff and patients. Stamps et al. (2021) also noted that nurses are rising to the occasion and exhibiting remarkable fortitude during turbulent times, and continue to put their lives on the line while caring for others. Stamps et al. (2021) reported that nurses need nurse leaders to advocate for their work conditions, safety, and welfare while they provide care under challenging conditions.

The ability to adapt to changing situations and effectively solve problems while fostering professional growth and providing mentorship opportunities was another recurrent theme in the current study. Tabloski (2016) reported that mentorship for new nurses is crucial to their success and retention as new employees. If we do not foster the growth and development of young nurses, they may flounder, become extremely frustrated, and seek out new alternative employment settings. Participants perceived mentorship as supporting professional development and empowerment among nursing staff. However, the present study did not assess whether mentorship resulted in improved retention, job satisfaction, nursing care, or patient outcomes (Bianchi et al. 2018).

The findings indicate that nurse managers adopt different leadership styles depending on the clinical context and organisational demands. Most participants reported using a mixed or situational leadership approach rather than relying on a single leadership style. These adaptive responses were mostly a response to resource constraints in their various settings ranging from financial, infrastructural, logistical, and human resource challenges. Leaders in a resource constraint environment must continually adjust to accommodate the changing circumstances rather than relying on standardised management and leadership approaches. The engagement in adaptive leadership by promptly modifying strategies to respond to emerging problems does not only help to resolve resource constraint difficulties but also creates an environment of trust and confidence in leadership. The laissez‐faire leadership style, however, was mentioned as sometimes adopted by leaders in certain situations. Several studies have reported that the most practised nurse leadership style is transformational (Moon et al. 2019; Asiri et al. 2016; Fischer 2016). Subordinates tend to feel comfortable in their working environment when they perceive supervisor support as a specific behaviour within transformational leadership (Lin et al. 2015). In a study to identify significant differences in nursing leadership strengths by position title, Herman et al. (2015) reported that nursing positions at the director level and above were strongest in transformational leadership practices, while those at the manager level and below were identified as needing additional leadership development. Ferreira et al. (2018) in a study at a university hospital located in the state of Bahia also noted that the practice of transformational leadership was identified frequently among nurses; however, they had difficulties in exercising this leadership model because of a lack of institutional support, since vertical leadership is the most adopted style, as well as a lack of training for care nurses, and weaknesses in communication and discussion of problems before decision making.

In Ghana, Aberese‐Ako et al. (2018) reported, after studying leadership styles in two Ghanaian hospitals in a challenging environment, that most managers practised transformational or transactional leadership; however, some leaders adopted a laissez‐faire approach at times due to challenges. They resorted to laissez‐faire leadership when they felt most powerless, especially regarding staff discipline and accountability, where the centralisation of the power to hire and fire severely limited their ability to exercise transactional leadership. Ofei and Paarima (2022) also reported that nurse leaders exhibited different leadership styles depending on circumstances, sometimes using a participative style, which was sometimes confused with the democratic style, followed by transformational and transactional leadership styles. Similarly, Asamani et al. (2016) noted that nurse managers used different leadership styles depending on the situation, but were more inclined toward supportive leadership, followed by achievement‐oriented and participative leadership.

The study's findings indicated that democratic leadership directly and positively influences nurses' engagement, collaboration, and job satisfaction. This leadership style ensures an inclusive environment where team members feel recognised and are encouraged to share their insights or ideas. These findings support Wong and Cummings (2007), who demonstrated that democratic leadership in the healthcare environment promotes communication and teamwork, enhancing patient outcomes.

Moreover, the participatory nature of democracy was revealed to motivate nurses to take ownership of their responsibilities and, eventually, foster innovation and a sense of purpose in their roles. The study also indicated that the democratic leadership style may be less effective in high‐pressure or emergencies, where swift decision‐making is crucial. This observation is consistent with Luthans et al. (2008), who posit that while democratic leadership promotes collaboration, its impact on group input can delay critical decision‐making in urgent situations.

In contrast, autocratic leadership was found to be both beneficial and challenging, depending on the environment. While it is sometimes perceived negatively due to its authoritative and restrictive nature, participants acknowledged its usefulness in crisis management where swift and decisive action is required. Alkuwari supports this finding from their study, emphasising that in high‐stakes environments, autocratic leadership provides the clarity and control essential to handling complex situations effectively (Alkuwari 2025).

However, the research's findings highlighted that prolonged use of this leadership style can lead to reduced job satisfaction, increased staff turnover, and a sense of disempowerment. The findings also concurred with those of Abdollahzadeh et al. (2017), who indicated that although this style of leadership ensures operational efficiency in emergencies, it often stifles creativity and limits team members' professional growth.

These findings underscore the importance of adapting leadership styles to specific environments within nursing practice. While the democratic style fosters collaboration and engagement, the autocratic leadership style is indispensable in critical situations which require swift decision‐making.

Similar to our findings, Ofei and Paarima (2022) noted that democratic, mixed, transformational, and transactional leadership approaches promoted nurses' intention to stay, accounting for 20.9% of the variance in intention to stay among nurses. Also, a cross‐sectional study among employees who have worked for the Ghana Health Service in Ghana concluded that transformational nurse leaders' ability to instill a feeling of self‐worth in their followers is the key motivator for them to commit to a certain performance goal; this boosts their followers' motivation and self‐efficacy, which aim to increase their productivity (Kwarteng et al. 2024).

Previous studies have demonstrated that leadership practices strongly influence employee performance and the work environment; hence, it is conceivable to believe that nurse manager leadership style contributes immensely to the nurse's organisational performance, quality of service to the patient, and the working environment of the employee (Deriba et al. 2017). It has been noted that leader‐empowering behaviour is linked to psychological empowerment and is therefore positively associated with improved outcomes (Elsetouhi et al. 2018). These findings suggest that strengthening nurse managers' leadership competencies may be a potential strategy for supporting healthy work environments; however, its effects on patient care outcomes require further empirical evaluation. The focus on the nurse manager's leadership style, therefore, is a logical and vital process to ensure nurses thrive in a healthy work environment. Alrobai (2020) indicates that efficient leadership styles and high‐quality leadership skills by the nurse manager contribute significantly to promoting excellent patient care, a healthy work environment, and nurse retention. Although numerous leadership theories have been proposed in previous studies, few align directly with Herzberg's Two‐Factor Theory. He developed the motivation‐hygiene factors which either determined job satisfaction or dissatisfaction in a work environment. He considered that while motivational factors lead more directly to job satisfaction in a work environment, hygiene factors can be unpleasant and lead to job dissatisfaction in a work environment. Motivational factors are instruments or elements which help or improve job satisfaction among employees, factors such as professional development opportunities, recognition, personal achievement and others, while hygiene factors include lack of basic conditions of service or work for employees. Accordingly, this study explored leadership practices through the lens of Herzberg's Two‐Factor Theory to examine how motivation and hygiene factors influence workplace outcomes and the creation of a healthy work environment.

A study conducted in Northern Italy examined nurses' job satisfaction based on their perceptions of nurse managers' leadership style and found that nurse managers who used transformational leadership had a strong positive correlation with achieving organisational goals through a healthy work environment for nurses (Morsiani et al. 2017). Similar findings were also observed in studies conducted in hospitals where nurse managers adopted a transformational leadership style (Casida and Parker 2011; Abdelhafiz et al. 2015). Morsiani et al. (2017) also reported that the transactional leadership style had the lowest correlation with nursing staff satisfaction. Conversely, in a study conducted in Jordan to explore how the leadership style of nurse leaders affects job satisfaction among nurses working in government and private hospitals, findings revealed that the transactional leadership style had a stronger relationship with job satisfaction than the transformational leadership style (Abdelhafiz et al. 2015). The stronger relationship was mainly influenced by a contingent‐reward style of leadership, in which nurse managers rewarded nurses meaningfully for task completion (Abdelhafiz et al. 2015). Studies have also illustrated that hospitals with strong financial reward systems tend to have motivated staff and better job outcomes (Lambrou et al. 2010). Thus, it is essential to note that, despite mixed (situational), democratic, autocratic, laissez‐faire, and transformational leadership approaches being highly regarded as better leadership styles, the effective application of leadership skills in motivating staff to achieve the shared vision is critical (Casida and Parker 2011).

Participants in this study reported organisational support, high workload, time constraints, implementation difficulties, communication challenges, and hierarchical structures as challenges encountered in implementing the mentioned nurse leadership styles. Algunmeeyn et al. (2024) in a study to identify barriers to effective clinical nursing leadership in Jordanian hospitals from the perspectives of nurse managers reported that power differentials, inconsistent connectedness with physicians, lack of early socialisation experiences, and clinical practice reform were barriers to the practice of nurse leadership. Alenezi (2022) also explored staff nurses' perceptions of the barriers to and facilitators of effective nurse leadership at a major Saudi hospital and noted that barriers to effective leadership include inadequate leadership education and skills development, limited authority and clinical empowerment, unawareness of the need for change, and poor communication.

These findings are consistent with those reported by Agyeman‐Prempeh et al. (2021), which focused on the challenges of nursing leadership in Ghana, stating that the unavailability of adequate funds and organisational challenges are constraints to the effective practice of nursing leadership. The majority of nurse leaders do not have a say in budgetary allocations for their units.

Consistent with the findings of the current study, Abdulsalam et al. (2018) identified limited awareness of transformational leadership concepts and inadequate access to leadership education and training as significant barriers to the effective implementation of transformational leadership.

Alenezi also reported that engaging with and listening to staff, seeking their ideas, recognising staff performance, and using motivational strategies are among the facilitators of nurse leadership style practices (Alenezi 2022).

The findings of this study indicate that facilitating open and transparent communication is good for effective nursing leadership. This concurs with a study conducted by Clavijo‐Chamorro et al. (2022), which reported that increased communication between nurse managers and the rest of the healthcare team helped them overcome some of the problems they encountered. A good relationship between nurse managers and staff nurses tended to result in feedback on ongoing developments from all team members, allowing obstacles related to leadership style practices to be addressed. Authentic leaders display fairness, justice, empathy, respect, trustworthiness, reliability, and believability (Hilson 2018). Specchia et al. (2021) also suggested that leaders who focus on transparency, self‐awareness, and promotion of a “work ethic” can empathise with their subordinates by recognising and understanding their concerns, needs, and desires.

In discussing the role of a supportive and inclusive culture in facilitating barriers to nurse leadership practices, Murray et al. (2018) reported that nurse leaders' employee engagement helps develop trust in leaders. Trust fosters a safety culture, and the visibility of leaders reinforces it. With the establishment of trust, organisational staff believe concerns will be heard and that the necessary patient safety changes will occur. Open communication channels built on trust lead to a no‐blame culture (Hu 2022). Hu (2022) suggests that organisations that have created a no‐blame safety culture achieve better patient outcomes. These outcomes occur when leaders create an environment in which staff are encouraged to report errors, adverse events, near misses, and unsafe practices so that system changes can be made (O'Connor and Carlson 2016).

## 5 Strength and Limitation

This study provides in‐depth insights into nursing leadership practices through qualitative interviews with experienced nurse managers. However, the findings are limited to three public healthcare facilities who make up a significant part of the healthcare delivery system in the Kumasi Metropolitan Area. The findings also reflect only nurse managers' perspectives, and may not have a direct cause and effect relationship to other nurse managers or nurses who are not in leadership position. Which may limit transferability to other healthcare settings or stakeholder groups.

## 6 Conclusion

This study explored nurse managers' perceptions of leadership practices within selected healthcare facilities in Ghana. Participants described nursing leadership in terms of supervision, effective communication, professional development and mentorship, motivation, participatory decision‐making, and the use of different leadership approaches according to contextual demands. A mixed or situational leadership approach was commonly described, alongside democratic, autocratic, laissez‐faire, transformational, transactional, and other approaches.

Participants also described organisational and contextual challenges, including workload, staffing limitations, resource constraints, hierarchical structures, and communication difficulties, which shaped how leadership practices were enacted. The findings therefore suggest that nurse managers perceive leadership practice as a context‐dependent process involving both interpersonal practices and organisational conditions.

These findings should be interpreted as nurse managers' perceptions and experiences rather than evidence of causal effects. The study does not establish that particular leadership practices improve patient outcomes, staff retention, job satisfaction, or workforce performance. Future research using longitudinal, intervention‐based, or multi‐stakeholder designs could examine whether and under what conditions the leadership practices identified in this study are associated with measurable organisational and workforce outcomes.

## 7 Implication for Nursing Management

### 7.1 Nursing Management

The findings suggest that structured leadership development programmes may be considered to strengthen nurse managers' competencies in adaptive leadership, communication, decision‐making, conflict resolution, and participatory management. Such programmes should be implemented where appropriate and evaluated to determine their feasibility, acceptability, and effectiveness in clinical settings.

Second, the prominence of mentorship in participants' accounts suggests that formalised mentorship and coaching frameworks may be considered as potential strategies for supporting professional development and leadership succession. Their effects on staff development, job satisfaction, retention, and other relevant outcomes should be evaluated in future implementation studies.

Finally, participants identified inadequate staffing, high workload, limited resources, and organisational constraints as barriers to effective leadership. These findings suggest that organisational support interventions, including workforce planning, adequate staffing, resource provision, and clear reporting structures, may be considered as potential strategies to facilitate effective nursing leadership. However, the effects of such interventions on leadership effectiveness, staff satisfaction, retention, the work environment, and patient care outcomes require empirical evaluation.

### 7.2 Education and Research

The findings of the study identified a gap in clinical nursing leadership development through the review of relevant literature, the literature review identified that though a lot of studies exist on nursing leadership it mostly pertains to nursing education in the classroom and little attention has been paid to leadership in the clinical sector. Future clinical‐based studies on enhancing the leadership practices of nurses in the clinical sector should be encouraged to enhance the level of leadership knowledge and skills of nurse managers.

Finally, the identified barriers, particularly high workload, inadequate resources, and lack of formal leadership training, indicate the need for policy‐level interventions through education and research. Health authorities and regulatory bodies may consider strengthening leadership education and continuing professional development for nurse managers, with future studies evaluating the feasibility and effectiveness of such interventions across different healthcare settings.

## Author Contributions

**Prosper Manu Abdulai:** project administration, validation, investigation, writing – review and editing, formal analysis, visualisation, resources, data curation, software, conceptualization. **Emmanuel Obromah Amoh‐Kusi:** conceptualisation, writing – review and editing, investigation, writing – original draft, formal analysis, methodology, validation, resources, data curation, visualisation, software. **Victoria Bam:** supervision, validation, visualisation, project administration, resources, writing – original draft. **Collins Atta Poku:** validation, visualisation, project administration, resources.

## Funding

The authors have nothing to report.

## Disclosure

Generative Artificial Intelligence (AI) Use: No generative artificial intelligence tools were used in the design, conduct, data analysis, interpretation, or reporting of this research. The authors take full responsibility for the content of the manuscript.

## Ethics Statement

Ethical approval for the study was obtained from the Committee on Human Research, Publication, and Ethics (CHRPE) of Kwame Nkrumah University of Science and Technology (CHRPE/AP/148/24). Permission was also obtained from the management of the participating healthcare facilities before data collection commenced. Participation was voluntary, and written informed consent was obtained from all participants. Confidentiality and anonymity were maintained by assigning unique identification codes to participants instead of using their names. All audio recordings, transcripts, and related research materials were stored securely and were accessible only to the research team.

## Consent

All participants gave written informed consent.

## Conflicts of Interest

The authors declare no conflicts of interest.

## Supporting information


**Table S1:** Completed 32‐item COREQ checklist.

## Data Availability

The data supporting the findings of this study are available from the corresponding author upon reasonable request. The data are not publicly available because they contain information that could potentially compromise participant confidentiality.
