# Supplementary material for: Nursing Leadership Practices Among Nurse Managers in the Kumasi Metropolitan Area: An Exploratory Descriptive Qualitative Study
Source: Nurs Open. 2026 Aug 31;13(9):e70791. doi: 10.1002/nop2.70791 (PMC13530395; doi:10.1002/nop2.70791)
Supplement: Supplementary file 1 [file NOP2-13-e70791-s001.docx]

**Supplementary**

Table S1: Completed 32-item COREQ Checklist

| **No.** | **COREQ item** | **Information reported in the manuscript** |
| --- | --- | --- |
| **Domain 1: Research team and reflexivity** | | |
| 1 | Interviewer/facilitator | Two research assistants conducted the interviews. |
| 2 | Credentials | Both interviewers had a Master of Philosophy in Nursing. |
| 3 | Occupation | Research assistants/interviewers with training and experience in qualitative health research. |
| 4 | Gender | Not reported. |
| 5 | Experience and training | Both interviewers had experience in qualitative interviewing and received refresher training before data collection. |
| 6 | Relationship established | No prior supervisory, managerial, clinical, or personal relationship existed between interviewers and participants. |
| 7 | Participant knowledge of interviewer | The study purpose and procedures were explained to participants before interviews. |
| 8 | Interviewer characteristics/reflexivity | Researchers maintained reflexive notes to document assumptions, methodological decisions, and observations. |
| **Domain 2: Study design** | | |
| 9 | Methodological orientation/theory | Qualitative study using reflexive thematic analysis informed by a contextualist epistemological position. |
| 10 | Sampling | Purposive sampling was used to recruit nurse managers with relevant leadership experience. |
| 11 | Method of approach | Potential participants were identified through nursing administration and approached in person. |
| 12 | Sample size | Nineteen nurse managers participated; saturation was reached after 17 interviews and confirmed with two additional interviews. |
| 13 | Non-participation | Twenty-five nurse managers were approached; 19 participated and six declined. |
| 14 | Setting of data collection | Face-to-face interviews were conducted in private offices or quiet spaces within the healthcare facilities. |
| 15 | Presence of non-participants | Not reported. |
| 16 | Description of sample | Participants were nurse managers from three selected healthcare facilities, representing different professional grades and levels of clinical and management experience. |
| 17 | Interview guide | A semi-structured interview guide was developed from the literature, study objectives, and Herzberg’s Two-Factor Theory and was reviewed and pilot-tested. |
| 18 | Repeat interviews | No repeat interviews were conducted. |
| 19 | Audio/visual recording | Interviews were audio-recorded with participants’ consent and supplemented with field notes. |
| 20 | Field notes | Field notes documented contextual observations and non-verbal cues. |
| 21 | Duration | Each interview lasted approximately 30–45 minutes. |
| 22 | Data saturation | Initial saturation was observed after the 17th interview; two further interviews confirmed saturation. |
| 23 | Transcripts returned | All 19 participants reviewed their transcripts for accuracy. |
| **Domain 3: Analysis and findings** | | |
| 24 | Number of data coders | Not explicitly reported. |
| 25 | Description of coding tree | Initial codes were generated and related codes were grouped into potential themes; detailed coding tree not reported. |
| 26 | Derivation of themes | Themes were developed inductively from participants’ accounts and subsequently considered in relation to Herzberg’s Two-Factor Theory. |
| 27 | Software | NVivo was used to organise transcripts, manage codes, retrieve coded extracts, and support thematic development. |
| 28 | Participant checking | Participants reviewed their transcripts for accuracy; they did not review or validate the final themes or findings. |
| 29 | Quotations presented | Participant quotations are presented to support the reported themes and sub-themes. |
| 30 | Data and findings consistency | Findings are presented according to the themes and sub-themes identified through the analysis. |
| 31 | Clarity of major themes | Seven major themes and their associated sub-themes are clearly presented. |
| 32 | Clarity of minor themes | Sub-themes are presented under supervision, communication, professional development, leadership styles, motivation, organisational structure, and high workload. |
